# Effects of ketogenic diet on metabolic and cognitive‐behavioral outcomes in a mouse model of cerebral amyloid angiopathy

**DOI:** 10.1002/alz.089680

**Published:** 2025-01-03

**Authors:** Victoria Pulido‐Correa, Chana Vogel, Lauren Rosenberg, Bianca Echeverria, Iliana Uribe, Ariana Hernandez, Lisa S Robison

**Affiliations:** ^1^ Nova Southeastern University, Fort Lauderdale, FL USA

## Abstract

**Background:**

Cerebral amyloid angiopathy (CAA), characterized by the accumulation of amyloid protein in the cerebral vasculature, is highly prevalent in Alzheimer’s disease (AD) patients and, on its own, increases the risk of hemorrhagic stroke, cognitive impairment, and dementia. Currently, there are no effective ways to treat or prevent CAA. Ketogenic diet (KD), characterized by high‐fat, low‐carbohydrate, and moderate amounts of protein consumption, has gained considerable attention in recent years for its potential therapeutic use in patients with neurodegenerative diseases, including Alzheimer’s disease. Studies in AD rodent models have found that KD and/or ketogenic supplements attenuate cognitive‐behavioral impairments, neuroinflammation, and oxidative stress, reduce the accumulation of Aβ plaques, and enhance synaptic plasticity. However, it is unknown whether KD can similarly benefit individuals with CAA. This study, therefore, aimed to examine the potential therapeutic effects of KD in a transgenic mouse model of CAA.

**Method:**

Male Tg‐SwDI mice underwent a four‐month dietary intervention with either a standard rodent chow (macronutrient breakdown: 14.4% fat, 26.1% protein, and 59.5% carbohydrates) or ketogenic diet (macronutrient breakdown: 93.4% fat, 4.7% protein, and 1.8% carbohydrates) starting at ∼3.5 months of age. Body weight, caloric intake, and fluid consumption were assessed twice weekly throughout the duration of the experiment. Three months after diet initiation, glucose and ketone body levels were assessed to determine metabolic status, followed by a battery of behavioral tests to assess general activity levels, cognitive function, and anxiety‐related behaviors.

**Result:**

KD resulted in nutritional ketosis, attenuated body weight and adiposity, lowered fasting and fed blood glucose levels, improved glucose tolerance, increased activity levels (open field, Y‐maze), and enhanced spatial learning and memory (Barnes maze). Additionally, mice fed with KD exhibited a positive trend toward improved spatial working memory (Y‐maze for spontaneous alternation).

**Conclusion:**

Taken together, these findings provide compelling evidence that KD positively impacts cognitive performance and metabolic function, suggesting it may be a viable therapeutic option for CAA. Future research should explore the therapeutic potential of KD in females, as well as the diets' long‐term efficacy.

